# Epilepsy as the symptom of a spinocerebellar ataxia 13 in a patient presenting with a mutation in the *KCNC3* gene

**DOI:** 10.1186/s12883-023-03304-5

**Published:** 2023-06-26

**Authors:** Shao Li, Dandan Shang, Yanjiao Du, Yan Li, Ruihua Liu

**Affiliations:** Department of Neurology, Luoyang Central Hospital Affiliated to Zhengzhou University, NO. 288, Middle Zhongzhou Road, Xigong Square, Luoyang, 471000 China

**Keywords:** SCA13, *KCNC3* gene, Epilepsy, Heterozygous mutation

## Abstract

**Background:**

The spinocerebellar ataxias (SCAs) refer to a diverse group of neurodegenerative illnesses that vary clinically and genetically. One of the rare subtypes within this group is SCA13, caused by mutations in the *KCNC3* gene. Currently, the prevalence of SCA13 remains uncertain, with only a couple of cases being documented in the Chinese population. This study presented a case study of SCA13, where the patient exhibited clinical symptoms of epilepsy and ataxia. The confirmation of the diagnosis was done through Whole Exome Sequncing.

**Case presentation:**

Since childhood, the seventeen-year-old patient has not been capable of participating in numerous sporting activities and has experienced multiple episodes of unconsciousness within the last two years. The neurological evaluation showed a lack of coordination in the lower limbs. Cerebellar atrophy was detected through brain magnetic resonance imaging (MRI). The patient’s gene detection results showed that they exhibit a heterozygous c.1268G > A mutation in the *KCNC3* gene located at chr19:50826942. Antiepileptic treatment was promptly administered to the patient, and as a result, her epileptic seizures were resolved quickly. She has since remained free of seizures. After a one-year follow-up, there was no apparent improvement in the patient’s health status except seizure free, which may have worsened.

**Conclusion:**

The case study highlights the importance of actively combining cranial MRI with genetic detection in patients with ataxia of no known cause, particularly in children and young patients, to establish an possibly obvious detection. Patients who are young and have ataxia that is first accompanied by extrapyramidal and epilepsy syndromes should be aware of the potential of having SCA13.

## Background

Spinocerebellar ataxia (SCA) refers to a diverse collection of ataxic disorders that are neurodegenerative and inherited in an autosomal dominant manner [1]. The spinocerebellar ataxia is characterized by a usual mutation process where amplification of CAG repeat sequence in exons makes polyglutamine chains. This process leads to the acquisition of novel hazardous functions that may also be responsible for the identical clinical signs observed in several subtypes of SCA. SCA commonly presents with various clinical manifestations, such as cerebellar ataxia, cognitive impairment, peripheral neuropathy, optic atrophy, eye movement disorder, retinopathy, epilepsy, and extrapyramidal movement disorder, as reported [2]. Next-generation sequencing techniques have led to the identification of several new genes. One of these genes is the *KCNC3* gene, which is responsible for causing a distinctive form of SCA known as spinocerebellar ataxia type 13 (SCA13) [3]. Currently, there is limited information on the prevalence of SCA13, with only two families identified in Chinese populations. In this report, we discuss a case of SCA13 that is representative of the condition, with a mutation identified in the *KCNC3* gene. The participants provided informed and written authorization.

## Case Presentation

During a visit to our outpatient care neurology clinic, a 17-year-old woman was stated by her mother as having difficulty participating in various sports, including playing football and running, throughout her school years. The patient had 4 times of unconsciousness and inability to respond to calls within 2 years, accompanied by straightening and twitching of both lower limbs. No tonic-clonic seizures were seen in the upper limbs, and no eyes turned up, jaws closed, tongue biting, head and trunk distortion, incontinence, relieved in a few minutes each time, complained of a mild headache after consciousness became clear. There were no autoimmune diseases, neuropsychiatric, or hereditary in the family or personal medical history. During the neurological investigation, it was observed that the bilateral heel-knee-tibia tests were unstable and inaccurate. During a straight-line assessment, the body was found to be prone to falling and shaking. The Romberg test yielded a positive result, indicating an impaired sense of balance. However, there were no signs of meningeal stimulation, as the test yielded a negative outcome. The individual’s blood pressure was measured while lying down and was 106/73mmHg. When the patient stood up, their blood pressure was 110/70mmHg. The results of C-reactive protein, erythrocyte sedimentation rate, routine blood tests, and thyroid function showed no significant irregularities.

**Fig. 1:**
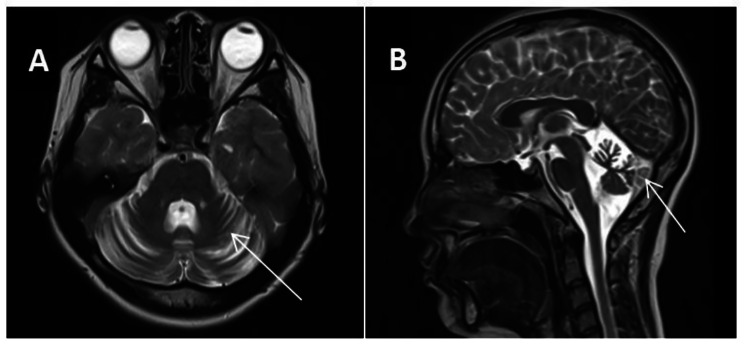
(**A**) Brain MRI axial sections demonstrated a considerable enlargement of the cerebellar sulcus (arrows); (**B**) Brain MRI sagittal sections demonstrated a clear atrophy of the cerebellum

Additionally, the patient tested negative for antibodies against HIV and Treponema pallidum particular antibodies. The MMSE Scale has a score range of 0–30 points, with a higher score indicating better cognitive function. The Montreal Cognitive Assessment (MoCA) Scale has a score range of 0–30 points, with a score of 26 or higher indicating normal cognitive function. The Activities of Daily Living (ADL) Scale has a score range of 0-100 points, with a higher score indicating greater independence in daily activities. In this case, the individual scored 30 on the MMSE Scale, 28 on the MoCA Scale, and 95 on the ADL Scale. The 24-hour dynamic electroencephalogram did not reveal any irregularities. The brain’s cerebellar atrophy is visible in the 1.5T MRI, as depicted in Fig. 1A and B.

**Fig. 2:**
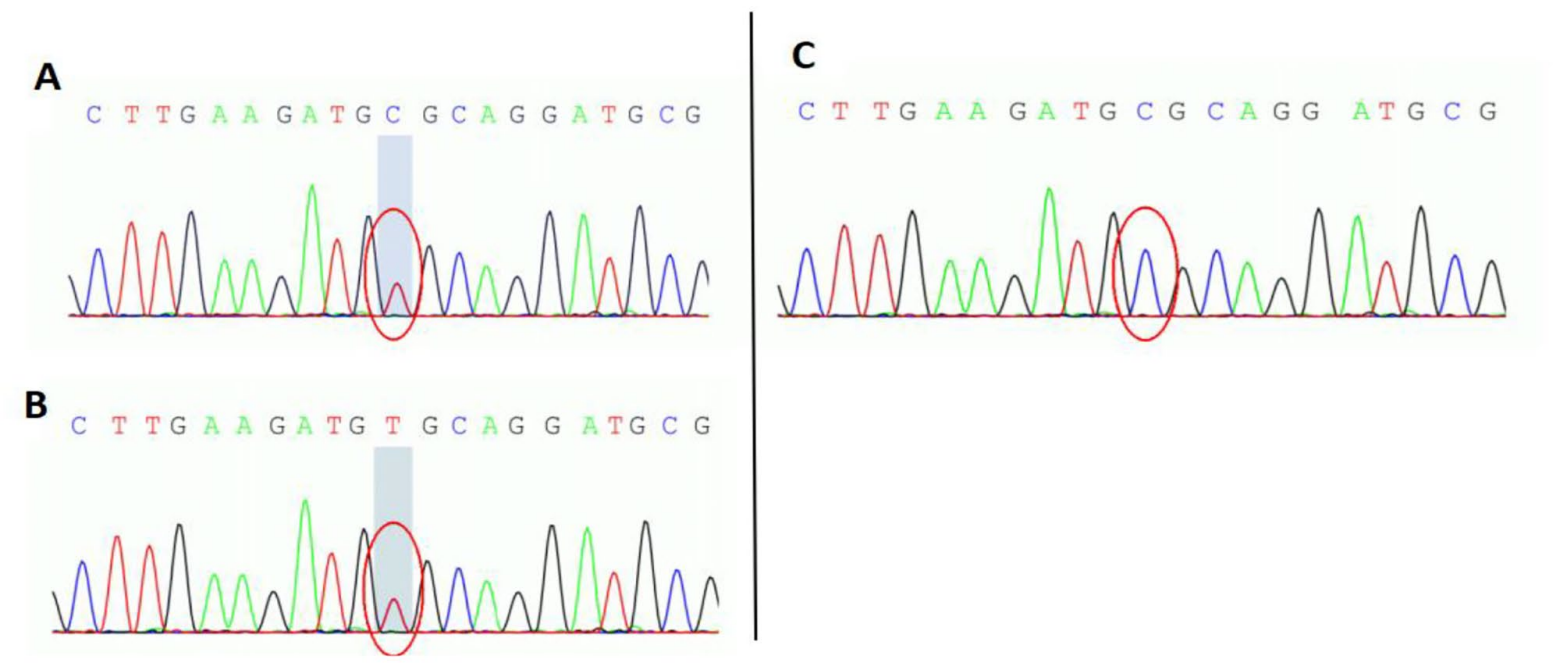
Displays the Sanger sequencing map of the *KCNC3* gene in patients and families. A red circle indicates the mutation site. (**A**) The patient’s *KCNC3* gene (located at chr19:50826942) exhibited a heterozygous variation of c.1268 G > A. (**B**) A heterozygous mutation of c.1268 G > A achr19:50826942 was also present in the patient’s mother. (**C**) Fater of the patient did not have any mutations

We diagnosed possible SCA based on these results. The patient’s *KCNC3* gene (located at chr19:50826942) was found to have the heterozygous mutation c.1268G > A (on the antisense strand, Fig. 2A) after the participant and her parents completed whole-exon genome sequencing. This mutation caused a substitution of histidine for arginine at location 423 (R423H). The variant in question has been categorized as a pathogenic variant (PS3 + PS4 + PM2_Supporting + PP3) based on the instructions provided by the American College of Medical Genetics and Genomics (ACMG). The family confirmation outcomes also indicate that the heterozygous mutation was inherited from the individual’s mother (Fig. 2B and C). The mother is currently not experiencing any clinical manifestations, but she would strongly resist undergoing a check-up for her convenience. While the patient was hospitalized, they received therapy in the form of coenzyme Q10 for nerve nutrition, citicoline capsules, and sodium valproate for antiepileptic purposes. Posture training has been found to enhance the function of balance. The patient’s symptoms were monitored for one year after discharge, and no significant modifications were observed.

## Discussion and conclusion

SCA has an estimated prevalence of 8–12 cases per 100,000 individuals. There are numerous genotypes associated with SCA, including the uncommon SCA13 form. SCA13 is characterized by a mutation in the voltage-gated potassium channel *KCNC3* (Kv3.3) on chromosome 19q13.3-19q13.4 and serves as the potential infectious gene for SCA13. *KCNC3* has six domains in its transmembrane (Supplementary files S1-S6). The Kv3.3 protein is found in significant quantities in cerebral Purkinje cells, neuronal cells in the spinal cord, and different brain stem parts. It is also present in the cerebral cortex and the hippocampus, although to a lesser extent [4, 5].

SCA13 was initially reported in families from the Philippines and France. Two different point mutations were found at 19q13.33, particularly F448L (c.1344 C > A) and R420H (c.1259G > A), according to the evaluation of the *KCNC3* sequence. The expression of R420H alone results in the abolition of channel activity. Moreover, when co-expressed with wild-type Kv3.3, it exerts an immense dominant adverse impact. On the other hand, F448L results in a shift of the activation curve toward the negative direction. It leads to a significant decrease in the rate of channel closure, which is around seven times slower than normal [3]. The expression of R420H results in alterations in the cell morphology and Golgi apparatus, subsequently impacting the expression of necessary proteins on the cell’s surface [6]. Research conducted on zebrafish pilots has revealed that mutations in Kv3.3 lead to alterations in the excitability of neuronal cells, which triggers the onset of SCA13 pathology [7, 8]. According to an investigation using the model of mice, the presence of R424H in cerebral Purkinje cells (PC) had a substantial effect on the activity of basal [Ca(2+)]I, the excitability of the neurons, and endogenous Kv3 channels. This ultimately resulted in the death of cells. The excitation of neurons may give rise to changes in calcium channels, which can result in cerebral nerve degeneration [9]. Hence, Patients with SCA13 exhibit clear cerebellar atrophy.

Currently, it exists numerous mutation locations associated with SCA13. In this case, the *KCNC3* gene carries the heterozygous mutation c.1268G > A, which causes a replacement of histidine for arginine at position 423 (R423H). To date, there have been more than 20 confirmed cases of SCA13 caused by unusual and classical alterations in the *KCNC3* gene [10]. There have been two observed SCA13 mutations in the Chinese population. One involved the R423H mutation, while another involved the c.1018G > A (p.Val 340 Met) mutation. The R423H mutation was initially identified as a pathogenic variant in the European population. The R423H and R420H mutations are situated in the S4 transmembrane region of the channel, which is known for its high level of conservation. A dominant negative mechanism suppresses Kv3 current amplitude through R423H and R420H [11]. The mutation in F448L bears a resemblance to the R423H mutation. SCA13 individuals are more likely to experience early onset due to the alteration of the gated channel region. On the other hand, the mutation in R420H results in a higher incidence of adult-onset. The ages at which symptoms begin largely depend on the changes in the ion-gated channel.

SCA13 is a condition that encompasses a range of symptoms, including non-progressive infantile-onset ataxia, as well as progressive childhood-onset and adult-onset cerebral ataxia. The age at which symptoms first appear can differ among individuals within a family and among different families, with some experiencing symptoms as early as twenty- two years old and others not until the age of sixty [13]. SCA13 exhibits varying clinical symptoms depending on the demographic group of the affected individuals. Infantile-onset ataxia is characterized by non-progressive ataxia in the trunk, limbs, or gait, tremors, dysarthria, and mild to moderately severe cognitive impairment. Participants may exhibit seizures, nystagmus, mental disturbance, and hyperreflexia. Diagnostic imaging proof indicates that cerebellar atrophy can occur well before the onset of clinical symptoms [14]. The onset of the disease in infants is typically marked by significant cerebral atrophy, ongoing motor difficulties, and intellectual impairment. On the other hand, the onset of the disease in adults is marked by gradually worsening ataxia and cerebellar deterioration. This information is supported by reference 9. Patients with F448H and R423H mutations display comparable clinical signs and symptoms, including movement disorders, seizures, and mental retardation. On the other hand, R420H exhibits clinical phenotypes identical to various SCAs, with symptoms progressing slowly, obvious limb and trunk ataxia, dysarthria, and gait instability [15]. The first account of cerebellar ataxia and seizures linked to R420H was published in 2013. The participant’s electroencephalogram (EEG) showed left mesial temporal (LMT) prominence and symptoms of left hippocampal sclerosis. The epileptic syndrome’s specifics are still not completely characterized, though [16]. Additionally, people with SCA13 may have sleep issues, uncontrollable trembling, and a toe-walking gait [17]. Throughout their childhood, the patient showed a typical level of intellectual growth. Ataxia symptoms were discovered when the person was a student, although they had little to no effect on their day-to-day activities. She then started having seizures, but her symptoms didn’t significantly improve despite obtaining therapy. The likelihood of non-progressive ataxia from infancy is thought to be quite high.

The primary therapeutic option for SCA13 remains empiric symptomatic management, complemented by extensive physical and rehabilitation therapies. There is, at present, no established treatment protocol for SCA13. Ataxia and its associated neurologic symptoms should be managed using a multidisciplinary strategy that involves professionals in behavioural/social concerns, educational requirements, feeding teams, speech and language pathology, occupational therapy, physical therapy, and neurology [12]. Currently, certain trials targeting specific subtypes have shown promising therapeutic results. According to a study, Acetazolamide effectively improves the manifestations of ataxia among individuals with SCA6 [18]. Umahara discovered that the relationship between ataxin-1 and 14-3-3 protein could be a promising candidate for a therapeutic approach in the management of SCA1 [19]. The effect of oligonucleotides with antisense (ASO) targeting Kv3.3 channels on mice with the Kv3.3-G592R coding mutation and wild-type mice was examined in this study. The results showed that inhibiting the expression of Kv3.3 had a positive effect on mice with the SCA13 mutation and minimal impact on the wild-type animals. Targeting the expression of Kv3.3 could be an effective therapeutic strategy for treating SCA13 [20].

## Discussion and conclusion

At present, there is a limited amount of research available on SCA13. Occasionally we come across patients with ataxia of unidentified origins, particularly those who developed it during childhood or young adulthood. It is important to thoroughly investigate their past medical conditions and perform an extensive physical and neurological evaluation. Once additional ataxia illnesses have been ruled out, it is recommended to conduct genetic testing to establish the medical diagnosis. Patients with ataxia who experience seizures and extrapyramidal syndrome as their first signs should consider their chances of having the disease.

## Data Availability

All data and material supporting our findings are contained within the manuscript.

## Abbreviations

SCA: Spinocerebellar ataxia
HIV: Human Immunodeficiency virus
MRI: Magnetic resonance imaging
MMSE: Mini-mental State Examination
MoCA: Montreal Cognitive Assessment
ACMG: American College of Medical Genetics
PC: Purkinje cells
ASO: Antisense oligonucleotides
